# Evidence of clinical efficacy and pharmacological mechanism of N-butylphthalide in the treatment of delayed encephalopathy after acute carbon monoxide poisoning

**DOI:** 10.3389/fneur.2023.1119871

**Published:** 2023-03-16

**Authors:** Huiping Song, Aochun Yue, Xudong Zhou, Wei Han, Qin Li

**Affiliations:** ^1^First School of Clinical Medicine, Shandong University of Traditional Chinese Medicine, Jinan, China; ^2^Emergency Department, Shenzhen University General Hospital, Shenzhen, China; ^3^Centre of Integrated Chinese and Western Medicine, School of Basic Medicine, Qingdao University, Qingdao, China; ^4^Department of Integrated Chinese and Western Medicine, Yuhuangding Hospital Affiliated to Qingdao University, Yantai, China

**Keywords:** network meta-analysis, network pharmacology, delayed encephalopathy after acute carbon monoxide poisoning, clinical efficacy, N-butylphthalide, pharmacological mechanism

## Abstract

**Objective:**

Based on network meta-analysis (NMA) and network pharmacology approaches, we explored the clinical efficacy of different regimens, and clarified the pharmacological mechanisms of N-butylphthalide (NBP) in the treatment of delayed encephalopathy after acute carbon monoxide poisoning (DEACMP).

**Methods:**

Firstly, NMA was conducted to obtain the ranking of the efficacy of different regimens for the treatment of DEACMP. Secondly, the drug with a relatively high efficacy ranking was selected and its mechanism of treatment for DEACMP was identified through a network pharmacology analysis. By the use of protein interaction and enrichment analysis, the pharmacological mechanism was predicted, and molecular docking was subsequently carried out to verify the reliability of the results.

**Results:**

A total of 17 eligible randomized controlled trials (RCTs) involving 1293 patients and 16 interventions were eventually included in our analysis from NMA. Mesenchymal stem cells (MSCs) + NBP significantly increased mini-mental state examination (MMSE) and Barthel index (BI) scores; NBP + dexamethasone (DXM) was the most effective treatment in improving the activity of daily living (ADL) scores; NBP significantly decreased national institutes of health stroke scale (NIHSS) scores; Xingzhi-Yinao granules (XZYN) had more advantages in improving Montreal cognitive assessment (MoCA) scores, translational direct current stimulation (tDCS) had a significant effect in improving P300 latency and P300 amplitude and Kinnado + Citicoline had the most obvious effect in improving malondialdehyde (MDA). Meanwhile, by network pharmacology analysis, 33 interaction genes between NBP and DEACMP were obtained, and 4 of them were identified as possible key targets in the process of MCODE analysis. 516 Gene ontology (GO) entries and 116 Kyoto Encyclopedia of Gene and Genome (KEGG) entries were achieved by enrichment analysis. Molecular docking showed that NBP had good docking activity with the key targets.

**Conclusion:**

The NMA screened for regimens with better efficacy for each outcome indicator in order to provide a reference for clinical treatment. NBP can stably bind *ALB, ESR1, EGFR, HSP90AA1*, and other targets, and may play a role in neuroprotection for patients with DEACMP by modulating Lipid and atherosclerosis, *IL-17* signaling pathway, *MAPK* signaling pathway, *FoxO* signaling pathway, *PI3K/AKT* signaling pathway.

## 1. Introduction

Delayed encephalopathy after acute carbon monoxide poisoning (DEACMP) is a pathological phase after the “false recovery period,” in which patients with acute carbon monoxide (CO) poisoning have no any clinical symptoms or the clinical symptoms have been significantly alleviated for several days or even weeks after treatment, and then they reappear neuropsychiatric symptoms dominated by dementia and motor disorder. The cerebral white matter's extensive demyelination in DEACMP is the primary pathogenic alteration, which is specifically manifested as cognitive impairment, slow response, dyskinesia, autonomic nervous system dysfunction, Parkinson's syndrome, and abnormal behavior or mobility disorder. The most frequent and efficient treatment for acute CO poisoning is hyperbaric oxygen therapy (HOT) (1–3), but it has been demonstrated that HOT cannot prevent the occurrence of neurological sequelae (4), and there is no specific treatment for DEACMP. Therefore, exploring more effective regimens is still the focus of current research. Network meta-analysis (NMA) and network pharmacology are research methods based on the concept of complex networks, the former is used to evaluate the therapeutic effects of multiple interventions, its greatest advantage is that all interventions in the same body of evidence are evaluated and ranked simultaneously (5), while the latter is characterized by “multi-gene, multi-target,” which reveals the mechanism of drugs by elaborating the complex network of drug-target-gene-disease relationships (6). Therefore, in the present study, we screened for regimens with better efficacy for each outcome indicator by NMA, and the pharmacological mechanism of N-butylphthalide (NBP), as a new neuroprotective agent, was then achieved and determined by network pharmacological analysis, so as to provide a better reference for clinical treatment and experimental studies.

## 2. Materials and methods

### 2.1. Network meta-analysis

#### 2.1.1. Search strategy and study selection

The Preferred Reporting Items for Systematic Reviews and Meta-Analyses (PRISMA) (5) for NMA guidelines were followed in the study. Literature search was conducted in PubMed, Web of Science, Embase, and Cochrane Library, the publication time was set from the establishment of the database to October 31, 2021. Using the following Medical Subject Headings (MeSH) terms and keywords: “delayed encephalopathy after acute carbon monoxide poisoning,” “carbon monoxide intoxication,” “acupuncture,” “mesenchymal stem cells (MSCs),” “mental cord blood mononuclear cells (CB-MNCs),” “nalmefene,” “n-butylphthalide (NBP),” “kinnado,” “Ginaton,” “Xingzhi Yinao granules (XZYN),” “glucocorticoids,” “dexamethasone (DXM),” “oxiracetam,” “translational direct current stimulation (tDCS),” “Citicoline,” “hyperbaric oxygen,” and other relevant conceptual keywords.

#### 2.1.2. Inclusion and exclusion criteria

Diagnostic Criteria for DEACMP (7). Any of the following clinical abnormalities observed 2–60 days after the recovery of the consciousness disorder caused by acute CO poisoning: (1) disturbances of mental state and/or consciousness (such as dementia or delirium), (2) extrapyramidal lesions (such as Parkinson's syndrome), and (3) pyramidal damage and focal cortical dysfunctions.

The inclusion criteria of the present study was set as follows: (1) Randomized controlled trials (RCTs); (2) Patients diagnosed with DEACMP; (3) Interventions included commonly used drugs for the treatment of DEACMP; and (4) At least one of the following outcomes was included: mini-mental state examination (MMSE), the national institutes of health stroke scale (NIHSS), the activity of daily living (ADL), Montreal cognitive assessment (MoCA), Barthel index (BI), P300 latency, P300 amplitude, malondialdehyde (MDA), and adverse reactions were measured with more than one outcome as their endpoints.

Exclusion criteria of the present study was: (1) Animal experiments; (2) Inconsistent intervention measures; (3) Duplicate articles; (4) Diagnostic or efficacy evaluation criteria were not mentioned; (5) There were obvious statistical errors in the literature; and (6) Research whose full text cannot be obtained.

#### 2.1.3. Literature screening and data extraction

According to the predetermined screening criteria, two researchers independently screened the papers, extracted data, and conducted cross-checks. Any disagreements among the researchers were discussed or arbitrated by a third party. Then articles were imported into Endnote, duplicates were removed, and preliminary screening was done by reading the titles and abstracts. If an article satisfied the requirements for inclusion, the reviewers carried out a full-text read. Finally, the first author, year of publication, sample size, age and course of the disease, intervention measures, outcome indicators, and course of treatment were mostly extracted using an Excel data extraction table.

#### 2.1.4. Quality assessment

The risk of bias (ROB) of the included studies was assessed using the Cochrane Risk of Bias tool. These studies were estimated from seven domains of ROB, defined as having a high, low, or unclear ROB, and the seven domains included “random sequence generation,” “allocation concealment,” “blinding of participants and personnel,” “blinding of outcome assessment,” “incomplete outcome data,” “selective reporting,” and “other bias.” The judgment of ROB was carried out by two authors separately in Review Manager (Version 5.3).

#### 2.1.5. Statistical analysis

The statistical method of NMA was based on the frequency framework, and all outcome indicators were analyzed by the random effect model. For continuous variables, the standardized mean difference (SMD) was used as the effect size; for dichotomous variables, the odds ratio (OR) was as the effect size and the corresponding 95% confidence interval (CI) was calculated. Stata 16.0 software was applied to select the frequencyological framework random-effects model for the NMA, and the data were pre-processed using the network group command, and inconsistency tests were to assess the consistency of the results of direct and indirect comparisons. Efficacy ranking was then performed and the figure of the surface under the cumulative ranking curve (SUCRA) was plotted. A SUCRA of 100% indicated that the intervention was absolutely effective, whereas a SUCRA of 0 pointed out absolutely ineffective (8). If a closed loop existed, an inconsistency test was performed to determine the degree of agreement between the results of direct and indirect comparisons. Finally, funnel plots were drawn to verify whether there were small sample effects in the network and whether there was publication bias in the included studies.

### 2.2. Network pharmacology

#### 2.2.1. Screening of drug-related targets

The two-dimensional (2D) chemical structure of the drug was downloaded in Spatial Data Format (SDF) from the PubChem database (https://pubchem.ncbi.nlm.nih.gov/). Then the SDF file was submitted to Pharmmapper (http://www.lilab-ecust.cn/pharmmapper/) database for target prediction. All targets were transformed into recognized gene symbols using the UniProt database (9) (http://www.Uniprot.org/), and complete protein names were corresponded to the gene abbreviations.

#### 2.2.2. Construction of drug-targets network

Excel was used to prepare the network and type files of drug and gene symbols, which were then imported into Cytoscape 3.9.2 to construct the drug-targets network.

#### 2.2.3. Screening of DEACMP-related targets

The GeneCards database (10) (https://www.genecards.org) and the Oline Mendeline Inheritance in Man (OMIM) database (11) (http://www.omim.org) were mined for potential targets related to DEACMP using the keyword “delayed encephalopathy after acute carbon monoxide poisoning.” The DEACMP-related targets were then obtained by integrating the targets from the two disease databases and removing duplicate values.

#### 2.2.4. Construction of protein-protein interaction network

The obtained drug targets and disease targets were intersected by Venny 2.1.0 (https://bioinfogp.cnb.csic.es/tools/venny/) software to obtain potential targets. Then the potential targets were submitted to the STRING11.0 database (12) (https://string-db.org) to construct a PPI network model, with the organism species set to “Homo sapiens” and all settings set to default. The CytoNCA (13) plugin in Cytoscape 3.9.2 was used to analyze the attribute values of each node in the intersection network. All nodes with degree values more than twice the median degree value were chosen as key targets. The PPI network was further analyzed by the Molecular complex detection (MCODE) (14) plugin in Cytoscape 3.9.2 to identify potential protein functional modules.

#### 2.2.5. Enrichment analysis

Gene ontology (GO) enrichment and Kyoto Encyclopedia of Genes and Genomes (KEGG) pathway enrichment analysis were performed by the Metascape database (15) (http://metascape.org/gp/index.html) to examine the biological function and potential mechanisms of the potential targets.

#### 2.2.6. Molecular docking

Molecular docking was performed among the drug and key target proteins. The three-dimensional (3D) structures of the key target proteins were downloaded from the Protein Data Bank (PDB) database (16) (https://www.rcsb.org). Then the key target proteins were imported into Molecular Operating Environment (MOE) software for removing water molecules and the original ligands, and molecular docking with the drug was then carried out. The binding activity is evaluated by the lowest binding energy.

## 3. Results

### 3.1. Network meta-analysis

#### 3.1.1. Characteristics of included studies

**Figure 1:**
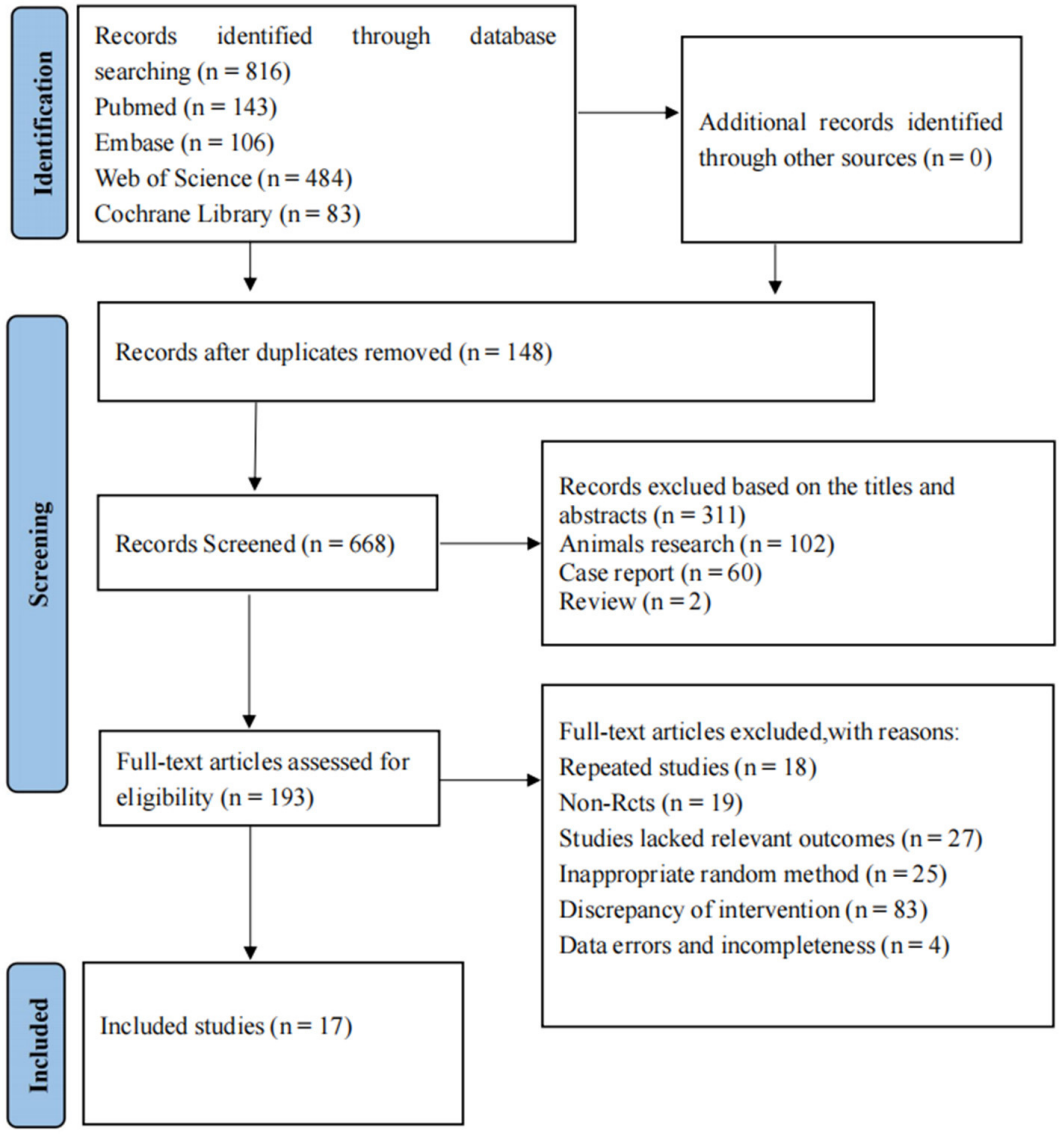
Literature screening flowchart.

A total of 816 articles were retrieved, and 17 articles were finally included after layer-by-layer screening (17–33), of which two articles were three-arm experiments and the other 15 were two-arm experiments. The literature selection process is shown in Figure 1. A total of 1,293 patients were considered eligible for this NMA, including 663 cases in the experimental group and 630 cases in the conventional therapy group. The basic characteristics of the included articles are shown in Table 1.

**Table 1:** Characteristics of included studies.

**References**	**Year**	**Age**		**Sample (M/F)**		**Interventions**		**Outcomes**
		**I**	**C**	**I**	**C**	**I**	**C**	
He (17)	2008	34.40 ± 16.80	32.40 ± 16.20	17/13	16/14	Acupuncture	C	②③
Liu et al. (18)	2013	56.30 ± 10.30	52.60 ± 9.90	21/11	20/10	Acupuncture	C	③
Yao et al. (19)	2014	42.37 ± 3.48	41.65 ± 3.07	19/11	18/12	CB-MNCs	C	①③
Mao et al. (20)	2015	58.00 ± 10.00	57.00 ± 11.00	10/11	8/12	Acupuncture	C	①⑤
Kong et al. (21)	2016	48.26 ± 10.48	45.36 ± 9.57	31/33	30/34	Nalmefene	C	①⑧
Wang et al. (22)	2016	56.00 ± 1.36	55.00 ± 1.69	8/6	8/7	MSCs+NBP	MSC	①③
			56.00 ± 1.41		7/6		C	
Wang et al. (23)	2016	56.62 ± 13.41	55.87 ± 14.16	NA	NA	Kinnado+Citicoline	Citicoline	⑧
			56.43 ± 14.35				C	
Wang et al. (24)	2017	47.40 ± 11.80	49.40 ± 11.30	28/30	32/26	Kinnado	C	①
Qin et al. (25)	2017	47.60 ± 8.20	48.50 ± 6.80	17/21	14/18	XZYN	C	①②③④
Xiang et al. (26)	2017	54.10 ± 12.00	53.50 ± 11.40	38/22	37/23	DXM	C	①②
Xiang et al. (27)	2017	52.35 ± 12.43	51.44 ± 10.82	52/42	49/41	NBP	C	①②
Sun et al. (28)	2018	46.60 ± 7.10	46.10 ± 8.10	14/13	13/12	Oxiracetam	C	⑥⑦
Li et al. (29)	2018	41.35 ± 5.26	42.03 ± 7.59	35/16	32/19	Hydrocortisone	C	①③
Zhang et al. (30)	2019	65.00 ± 5.00	62.00 ± 5.00	7/6	5/8	Acupuncture	C	①
Cao et al. (31)	2020	51.52 ± 6.21	53.65 ± 6.95	12/14	13/12	tDCS	C	①②⑤⑥
Lu et al. (32)	2020	50.60 ± 13.20	58.40 ± 11.80	7/7	6/8	Acupuncture	C	⑤
Zhang et al. (33)	2020	59.64 ± 12.28	58.59 ± 13.63	49/42	49/31	NBP+DXM	C	①③④

①, Mini-mental state examination (MMSE); ②, National institutes of health stroke scale (NIHSS); ③, activity of daily living (ADL); ④, Montreal cognitive assessment (MoCA); ⑤, Barthel index (BI); ⑥, P300 latency; ⑦, P300 amplitude; ⑧, Malondialdehyde (MDA). I, intervention group; C, conventional therapy group; NA, not available; CB-MNCs, mental cord blood mononuclear cells; MSCs, mesenchymal stem cells; NBP, N-butylphthalide; XZYN, Xingzhi Yinao granules; DXM, dexamethasone; tDCS, translational direct current stimulation.

The drugs used in the intervention group are all based on conventional treatment, which is abbreviated here and is only represented by the name of the drug.

#### 3.1.2. Quality assessment

**Figure 2:**
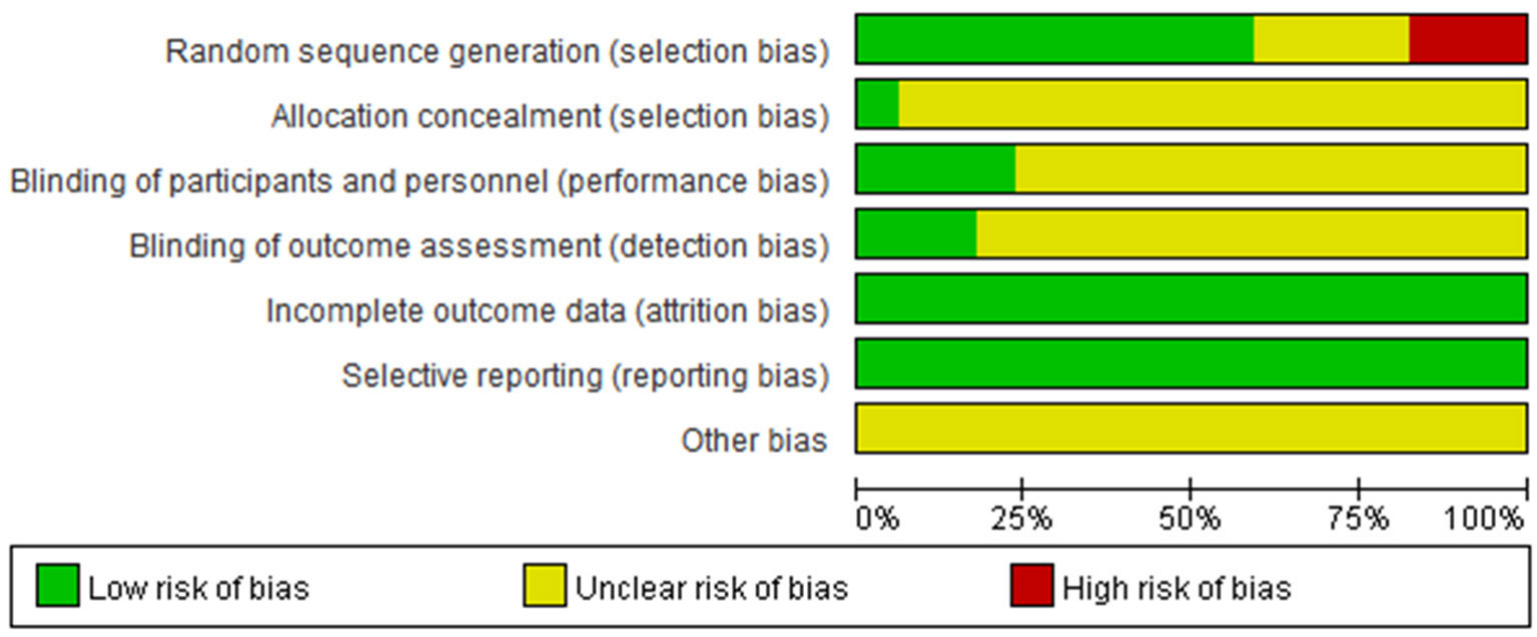
Quality assessment results.

All 17 studies were RCTs, the main risk of bias in this study came from “random sequence generation (selection bias),” 13 studies reported on the method of generating specific randomized sequences, four of them distributed treatment regimens according to the order of admission, therefore, it is defined as high risk. One study applied allocation concealment, four studies were blinded, all 17 studies reported on the outcome indicators expected to be measured and did not identify early termination of trials (Figure 2).

#### 3.1.3. Evidence network

**Figure 3:**
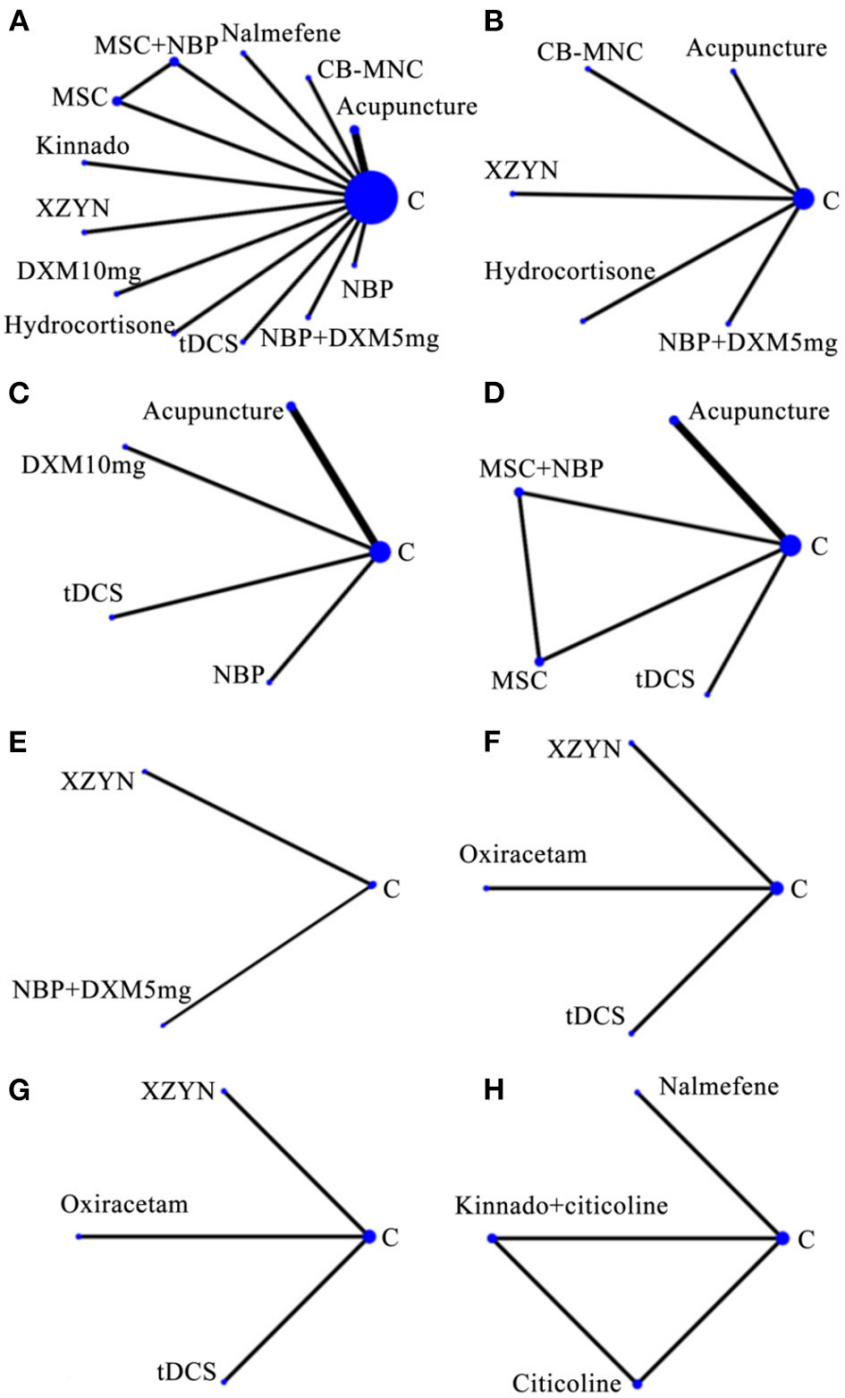
Network of evidence of included studies. **(A)** MMSE. **(B)** ADL. **(C)** NIHSS. **(D)** BI. **(E)** MoCA. **(F)** P300 latency. **(G)** P300 amplitude. **(H)** MDA. MMSE, mini-mental state examination; NIHSS, national institutes of health stroke scale; ADL, activity of daily living; MoCA, Montreal cognitive assessment; BI, barthel index; MDA, malondialdehyde; CB-MNCs, mental cord blood mononuclear cells; MSCs, mesenchymal stem cells; NBP, N-butylphthalide; XZYN, Xingzhi Yinao granules; DXM, dexamethasone; tDCS, translational direct current stimulation.

Network plots of outcome indicators were constructed to exhibit all the available evidence of each treatment. All network relationships were generally centered on Conventional Therapy treatment, the dot size represented the sample size of the intervention, and the line thickness represented the number of RCTs using that treatment (Figure 3).

#### 3.1.4. Inconsistency test

The closed loops in the reticulation were three-armed studies from the same article and there was no inconsistency.

#### 3.1.5. Network meta-analysis results

The league plots of meta-analysis and probability ranking results are displayed in Table 2. A total of 12 studies reported MMSE, including 13 interventions and producing 78 two-by two comparisons, and 50 of the comparisons were statistically significant (*P* < 0.05, Table 2A); 5 studies reported ADL, including six interventions and producing 15 two-by two comparisons, and 10 of the comparisons were statistically significant (*P* < 0.05, Table 2B); five studies reported NIHSS, including five interventions and producing 10 two-by two comparisons, and six of the comparisons were statistically significant (*P* < 0.05, Table 2C); four studies reported BI, including five interventions and producing 10 two-by two comparisons, and 9 of the comparisons were statistically significant (*P* < 0.05, Table 2D); two studies reported MoCA, including three interventions and producing three two-by two comparisons, and two of the comparisons were statistically significant (*P* < 0.05, Table 2E); three studies reported P300 latency, including four interventions and producing six two-by two comparisons, and three of the comparisons were statistically significant (*P* < 0.05), Table 2F); three studies reported P300 amplitude, including four interventions and producing six two-by two comparisons, and three of the comparisons were statistically significant (*P* < 0.05, Table 2G); two studies reported MDA, including four interventions and producing six two-by two comparisons, and five of the comparisons were statistically significant (*P* < 0.05, Table 2H). Significant pairwise comparisons were highlighted in TextTitle and underlined.

**Table 2:**
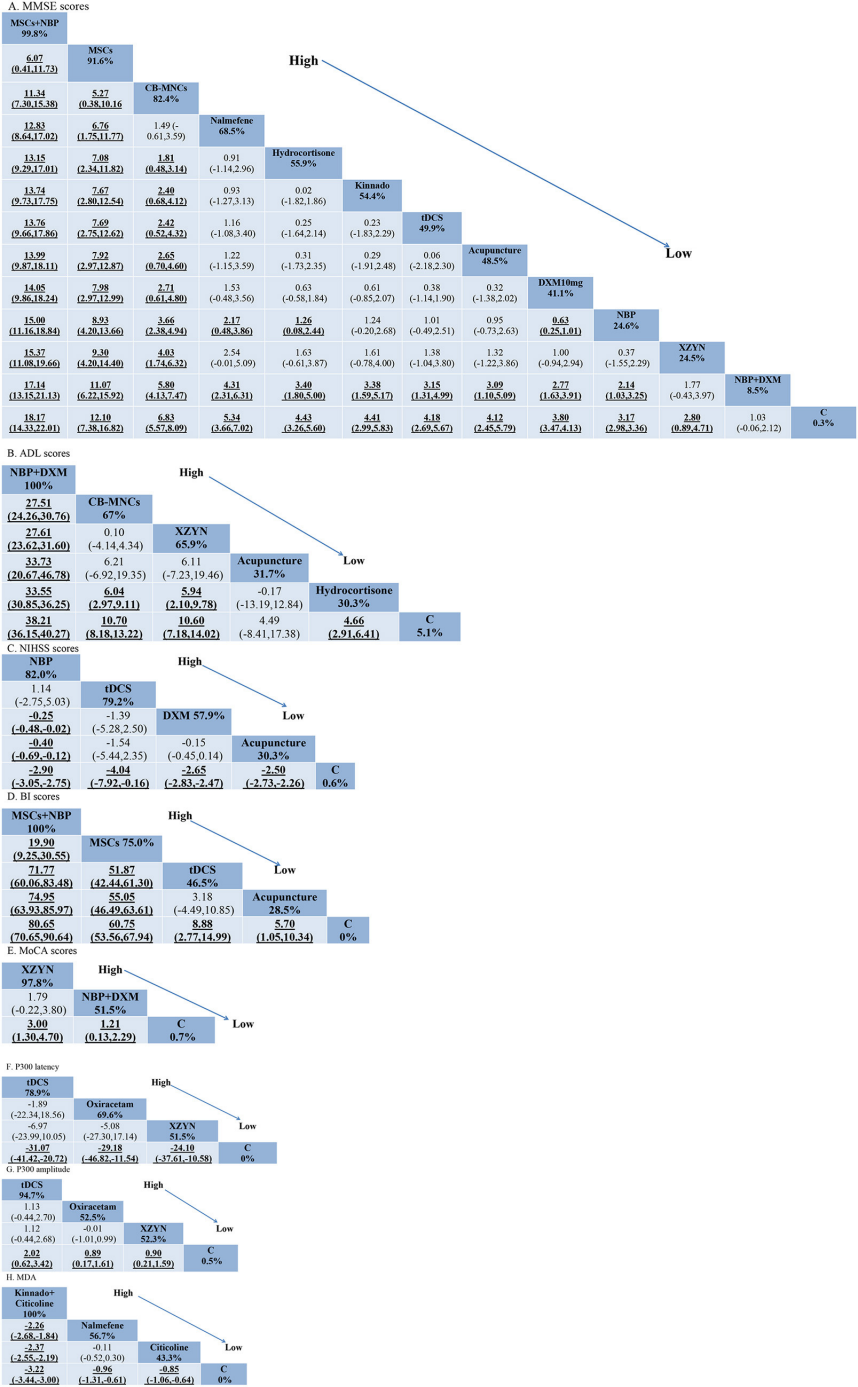
Relative effects of various outcomes [SMD (95% CI)].

Treatments are ranked according to their chance of being the best treatment. “High” means the highest probability of being the best treatment, and “Low” means the lowest probability of being the best treatment. Numbers in the dark blue boxes are the SUCRA values, the higher the value, the greater the probability of being the best intervention. Significant pairwise comparisons are highlighted in TextTitle and underlined.

MMSE, mini-mental state examination; NIHSS, national institutes of health stroke scale; ADL, activity of daily living; MoCA, Montreal cognitive assessment; BI, barthel index; MDA, malondialdehyde; C, conventional therapy group; CB-MNCs, mental cord blood mononuclear cells; MSCs, mesenchymal stem cells; NBP, N-butylphthalide; XZYN, Xingzhi Yinao granules; DXM, dexamethasone; tDCS, translational direct current stimulation.

#### 3.1.6. SUCRA probability ranking results

The SUCRA results indicated that: the probability of SUCRA for MMSE scores was ranked from highest to lowest: MSCs + NBP, MSCs, CB-MNCs, Nalmefene, Hydrocortisone, Kinnado, tDCS, Acupuncture, DXM, NBP, XZYN, NBP + DXM, Conventional Therapy (where the dosage form of NBP differs between “NBP” and “NBP + DXM,” the former being NBP soft capsules and the latter being NBP injection; the doses of DXM before and after were different, the former was DXM 10 mg, the latter was DXM 5 mg), MSCs + NBP was probably the most effective intervention (Table 2A); the probability of SUCRA in ADL scores was ranked from highest to lowest as follows: NBP + DXM, CB-MNCs, XZYN, Acupuncture, Hydrocortisone, Conventional Therapy, NBP + DXM may be the most effective intervention (Table 2B); the probability of SUCRA for NIHSS scores was ranked from high to low as follows: NBP, tDCS, DXM, Acupuncture, Conventional Therapy, NBP may be the most effective intervention (Table 2C); the probability of SUCRA for BI scores was ranked from high to low as follows: MSCs + NBP, MSCs, tDCS, Acupuncture, Conventional Therapy, MSCs + NBP was probably the most effective intervention (Table 2D); the probability of SUCRA of MoCA scores was ranked from high to low as follows: XZYN, NBP + DXM, Conventional Therapy, XZYN may be the most effective intervention (Table 2E); the probability of SUCRA for P300 latency was ranked from high to low as follows: tDCS, Oxiracetam, XZYN, Conventional Therapy, tDCS may be the most effective intervention (Table 2F); the probability of SUCRA for P300 amplitude was ranked from high to low as follows: tDCS, Oxiracetam, XZYN, Conventional Therapy, tDCS may be the most effective intervention (Table 2G); the results of SUCRA probability for MDA was ranked from high to low as follows: Kinnado + Citicoline, Nalmefene, Citicoline, Oxiracetam, Kinnado + Citicoline may be the most effective intervention (Table 2H).

#### 3.1.7. Publication bias

**Figure 4:**
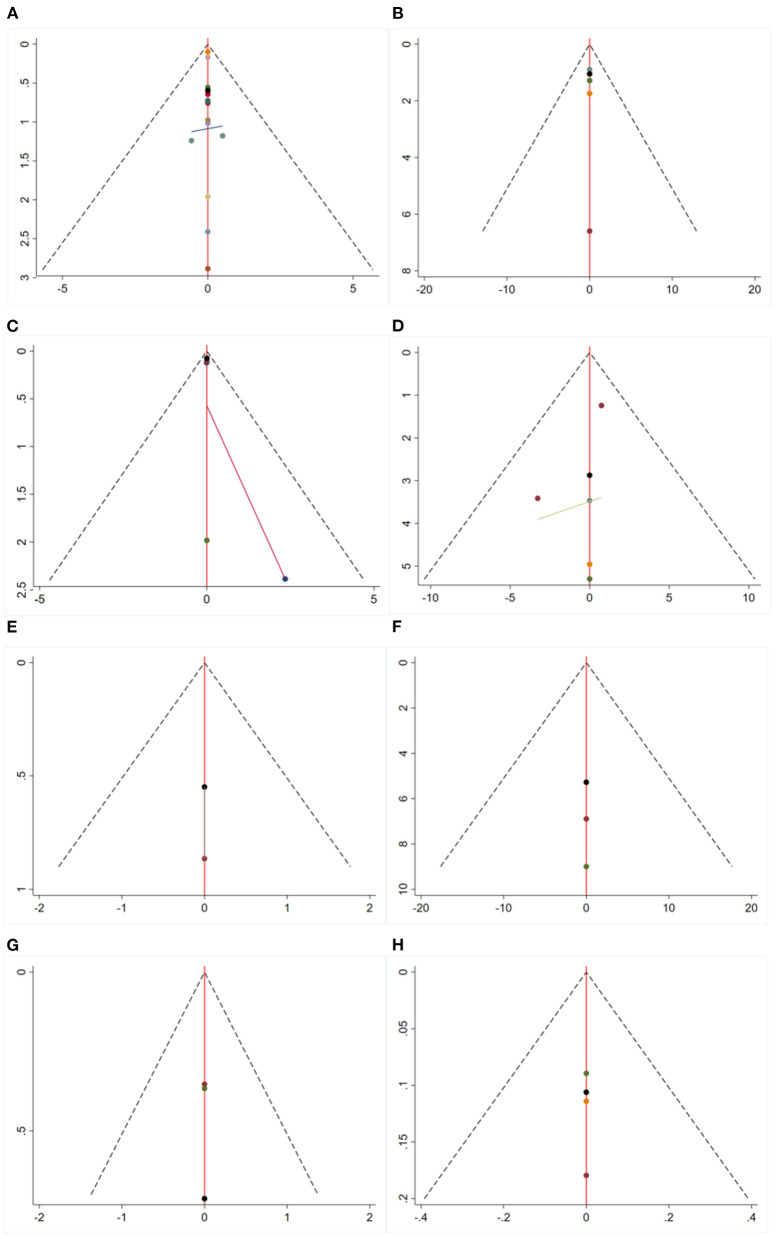
Funnel plots. **(A)** MMSE. **(B)** ADL. **(C)** NIHSS. **(D)** BI. **(E)** MoCA. **(F)** P300 latency. **(G)** P300 amplitude. **(H)** MDA. MMSE, mini-mental state examination; NIHSS, national institutes of health stroke scale; ADL, activity of daily living; MoCA, Montreal cognitive assessment; BI, barthel index; MDA, malondialdehyde.

The results of the inverted funnel plots showed a concentrated distribution of all studies in the interior of the “funnel,” the nodes symmetrically distributed on both sides of the axis, indicating a low likelihood of publication bias (Figure 4).

#### 3.1.8. Safety

Adverse reactions were reported in nine studies, while no serious adverse reactions were observed in all included studies. Adverse reactions that occurred during treatment period included hypothermia, dizziness, headache, swelling, pain in the ear, chest tightness, panic, and mild nausea, which recovered on their own without any specific treatment. Since most studies failed to report adverse reactions in a standardized manner, quantitative analysis of adverse reactions was not performed in this study.

#### 3.1.9. Shortcomings

This study had some limitations: (1) Some of the included studies did not report baseline data on patients comprehensively, such as the duration of disease and sex ratio of patients; (2) A rigorous exclusion of the literature was carried out to ensure homogeneity between studies, but there was still some clinical heterogeneity in the interventions of the control group in the included studies, such as the medication regimen, dose, frequency, and duration of medication. Meanwhile, sampling error, differences in patient age and gender, differences in underlying medical conditions, and differences in smoking and alcohol history were also sources of heterogeneity; (3) The disease is not frequently-occurring, and the number of RCT articles on this disease in the database is small, the number of included studies was only 17, and the literature of some treatment options had only one article, so the ranking of efficacy was not stable. Further validation with high-quality, multi-center, large-sample RCTs is recommended in the future.

### 3.2. Network pharmacology results

### 3.2.1. Prediction of targets associated with NBP

**Figure 5:**
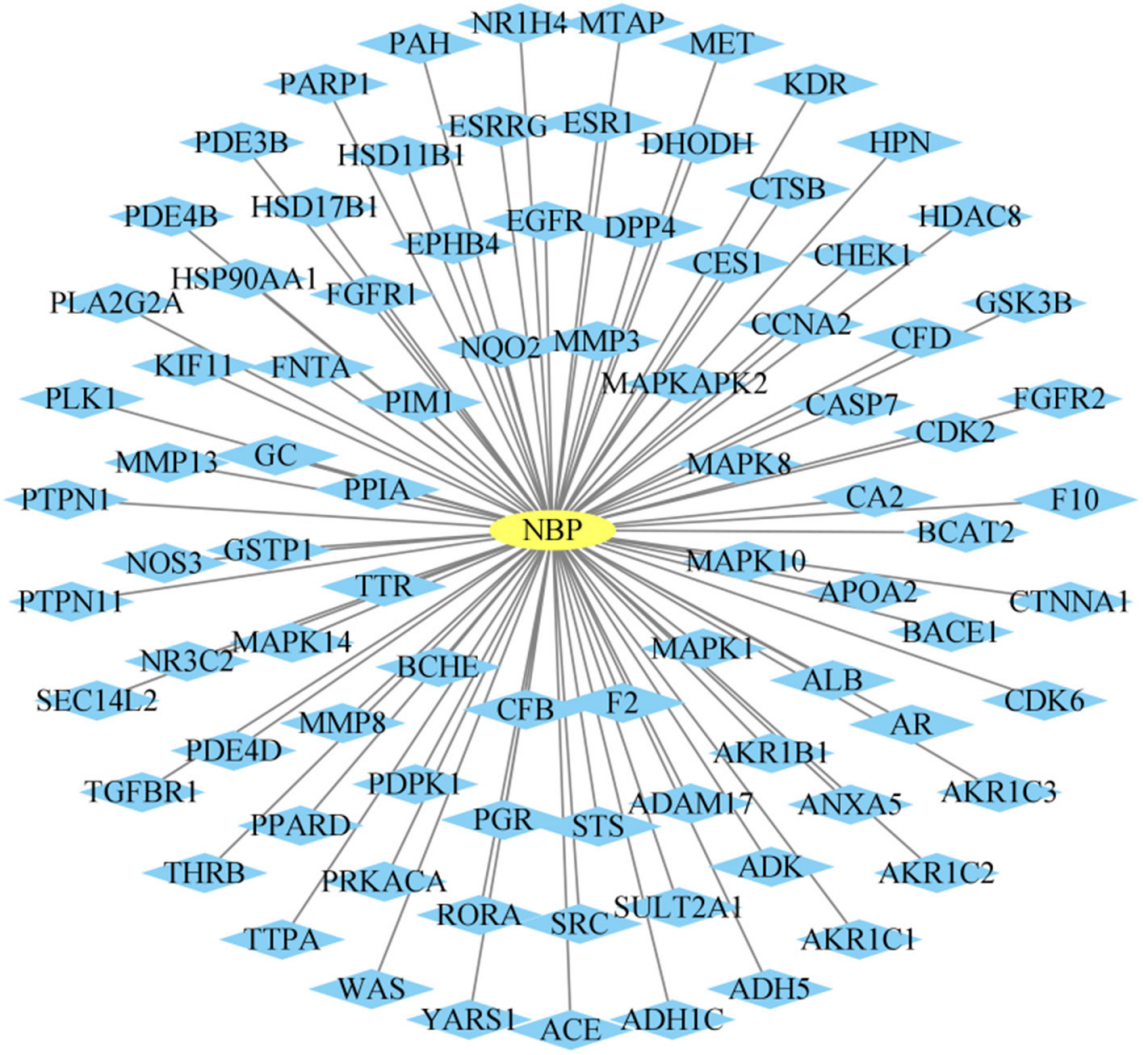
N-butylphthalide (NBP)-related target genes. The “ellipse” node represents NBP, the “diamond” node represents the potential therapeutic targets, and the edge represents the association between NBP and the potential target.

To further investigate the pharmacological mechanism of NBP, a network pharmacology analysis was performed. One hundred and thirty-seven predicted targets of NBP were obtained from the Pharmmapper database. Cytoscape 3.9.2 software was used to map and analyze the relationship network between NBP and its targets, as shown in Figure 5.

### 3.2.2. Prediction of targets associated with DEACMP

Five hundred and seventy-three DEACMP targets were obtained from the Genecards database and 618 DEACMP targets were achieved from the OMIM database, results were merged and the duplicate genes were eliminated, then 1154 DEACMP-related targets were identified.

### 3.2.3. PPI network

**Figure 6:**
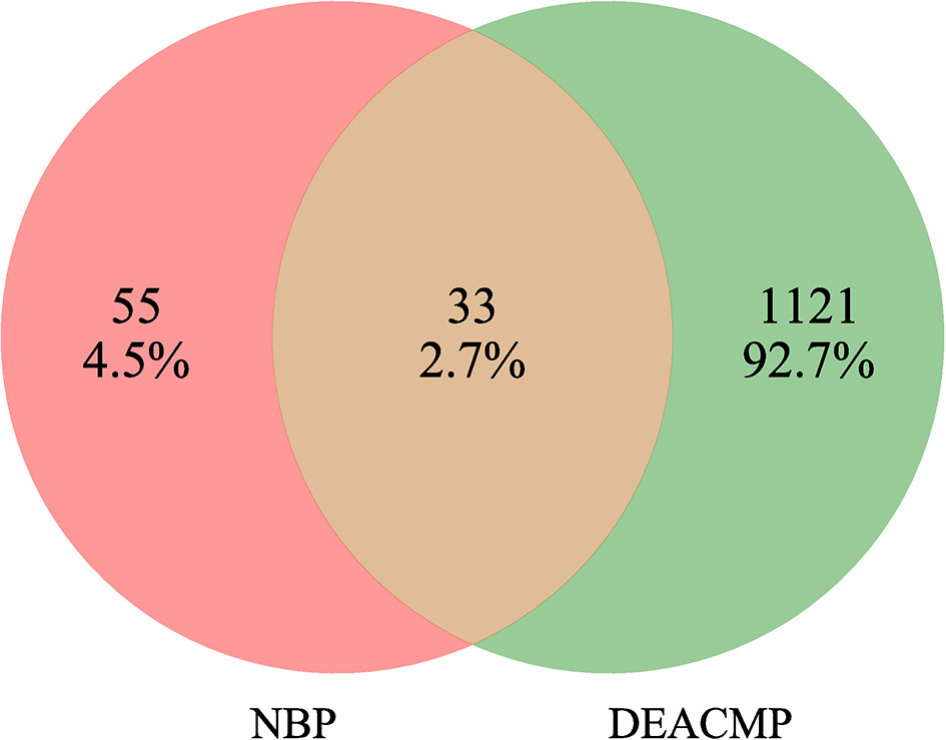
Venn diagram of genetic targets of N-butylphthalide (NBP) for the treatment of Delayed encephalopathy after acute carbon monoxide poisoning (DEACMP).

**Figure 7:**
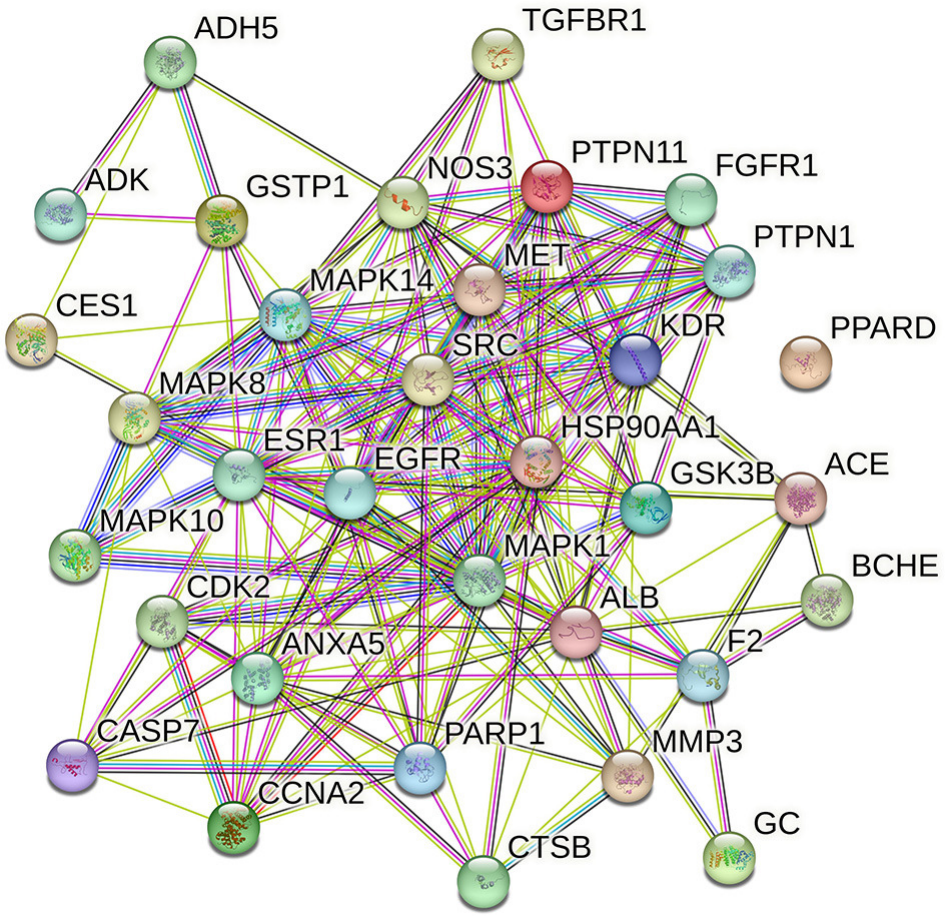
Protein-protein interaction (PPI) network diagram.

**Figure 8:**
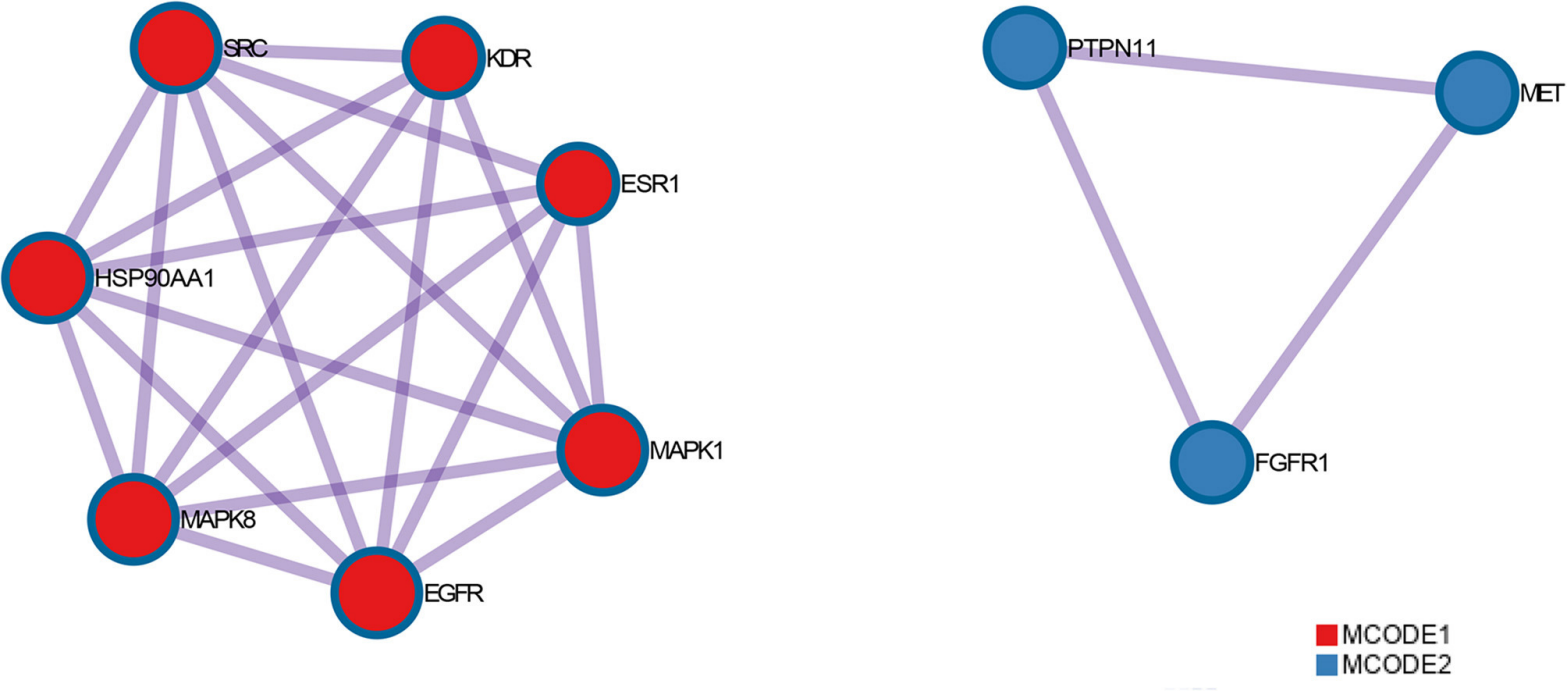
Modules in the protein-protein interaction (PPI) network.

NBP targets were intersected with DEACMP disease targets and 33 common targets for NBP-DEACMP were obtained by plotting Venn diagrams (Figure 6). The potential targets were submitted to STRING 11.0 for PPI network construction. The PPI network contains 33 nodes and 189 interactions (edges) between NBP and DEACMP (Figure 7). The results of the topological analysis showed that the average local clustering coefficient was about 0.694, the average degree of freedom was about 11.5, and there were 4 cases with degree values >2 times k1, which may be the key targets of NBP in the treatment of DEACMP (Table 3). Two protein functional modules were obtained by further analysis, one showing the relationship between *sarcoma (SRC), Heat Shock Protein 90*α *(HSP90AA1), mitogen-activated protein kinase 8 (MAPK8), epidermal growth factor receptor (EGFR), mitogen-activated protein kinase 1 (MAPK1), estrogen receptor 1 (ESR1), kinase domain receptor (KDR)*, and another showing the relationship between *protein tyrosine phosphatase nonreceptor 11 (PTPN11), fibroblast growth factor receptor 1 (FGFR1), mesenchymal-epithelial transition factor (MET)* (Figure 8).

**Table 3:** Basic information on key targets and molecular docking results.

**Gene**	**Full name of gene**	**Degree**	**PDB ID**	**Binding energy**
ALB	Albumin	27	4emx	−5.0205
ESR1	Estrogen Receptor 1	23	7ujm	−5.6201
EGFR	Epidermal growth factor receptor	22	7jxq	−5.1618
HSP90AA1	Heat shock protein 90α	21	3o0i	−5.5774

### 3.2.4. Enrichment analysis

**Figure 9:**
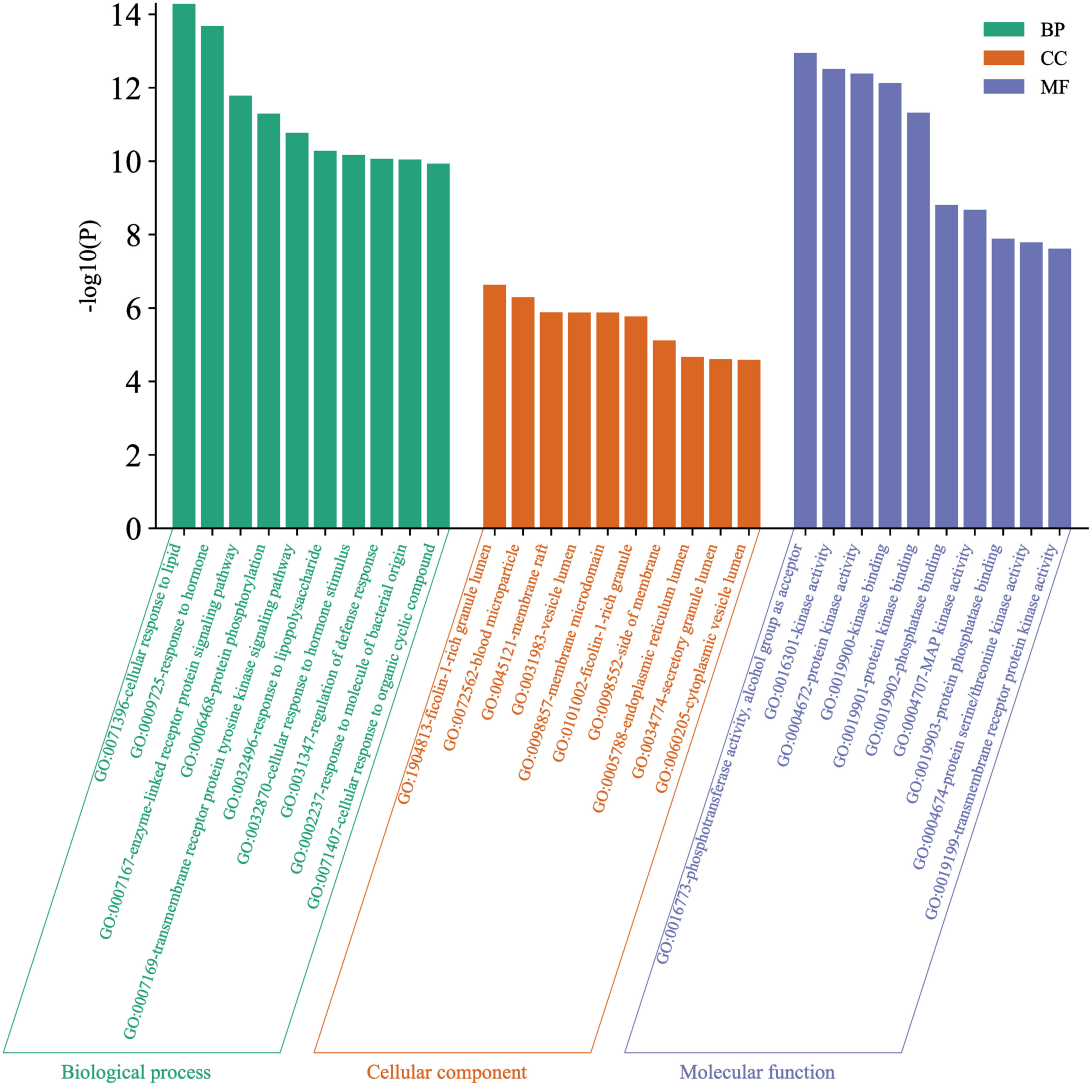
Gene ontology (GO) enrichment analysis. Green indicated biological process (BP), orange indicated cellular composition (CC), and purple indicated molecular function (MF).

**Figure 10:**
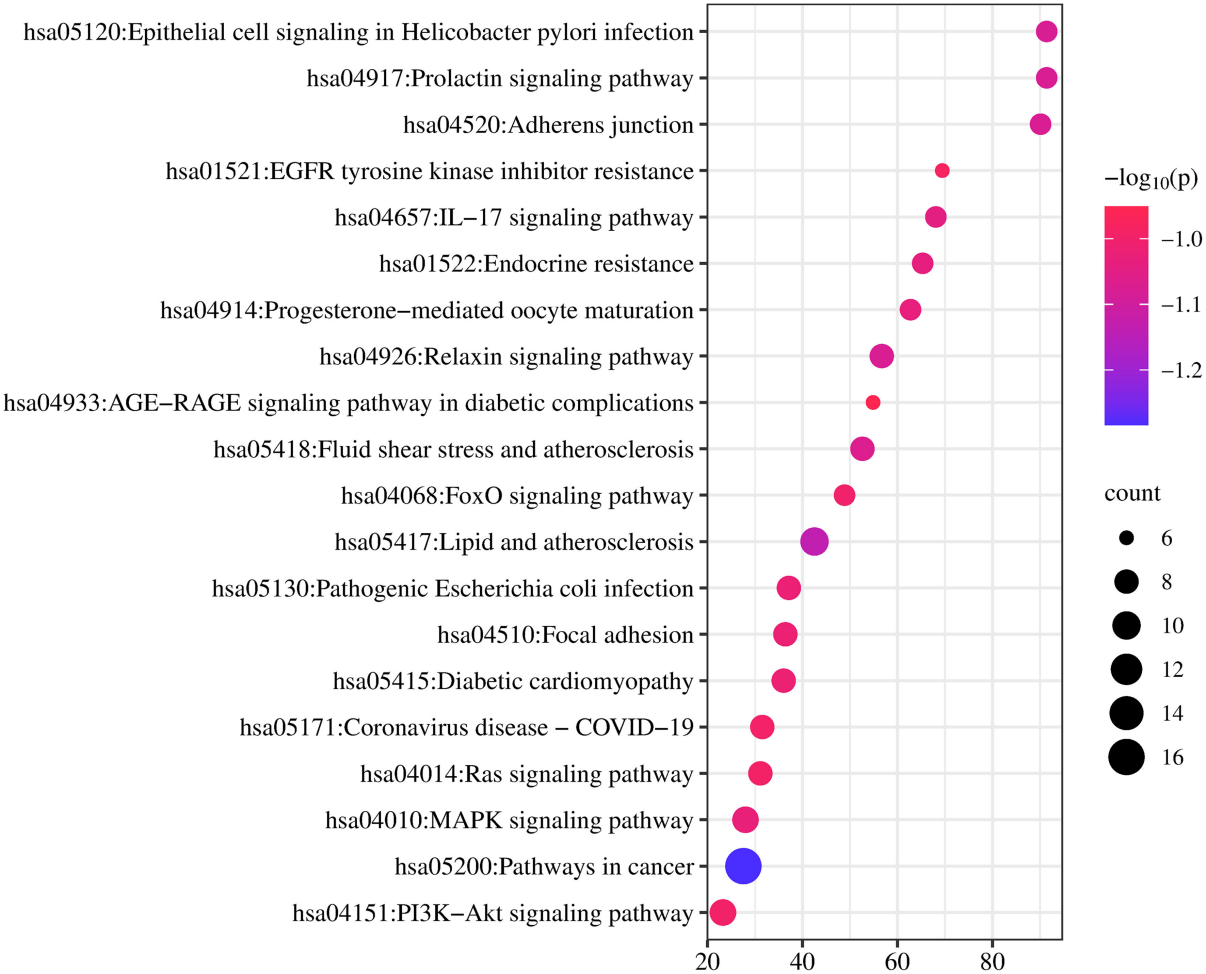
Kyoto Encyclopedia of Genes and Genomes (KEGG) enrichment analysis. The size of the dot reflected the change in the count. The larger the count, the larger the dot. The color reflected the size of the *P*-value, the larger the *P*-value, the darker the color.

GO and KEGG pathways enrichment analyses were performed to explore the potential functions of the 33 potential target genes. Five hundred and sixteen GO entries were screened out according to *P* ≤ 0.01. Among them, there were 436 biological processes (BP) entries, 37 cellular components (CC) and 43 molecular functions (MF). Figure 9 shows the top 10 enrichment pathways for the three entries above, respectively. There were 116 KEGG enrichment items in total, Figure 10 shows the top 20 items. In the BP category, the target proteins were mainly involved in the cellular response to lipids, response to the hormone, enzyme-linked receptor protein signaling pathway, protein phosphorylation, transmembrane receptor protein tyrosine kinase signaling pathway, etc. In the CC category, the target proteins were classified into the ficolin-1-rich granule lumen, blood microparticle, membrane raft, vesicle lumen, membrane microdomain, etc. In the MF category, the target proteins mainly participated in phosphotransferase activity, alcohol group as acceptor, kinase activity, protein kinase activity, kinase binding, protein kinase binding, etc. The most significant pathways included Lipid and atherosclerosis, *Interleukin-17 (IL-17)* signaling pathway, *MAPK* signaling pathway, *forkhead box O (FoxO)* signaling pathway, *phosphatidylinositol 3' -kinase (PI3K)/ protein kinase B (AKT)* signaling pathway, etc. (Figure 10).

### 3.2.5. Molecular docking

**Figure 11:**
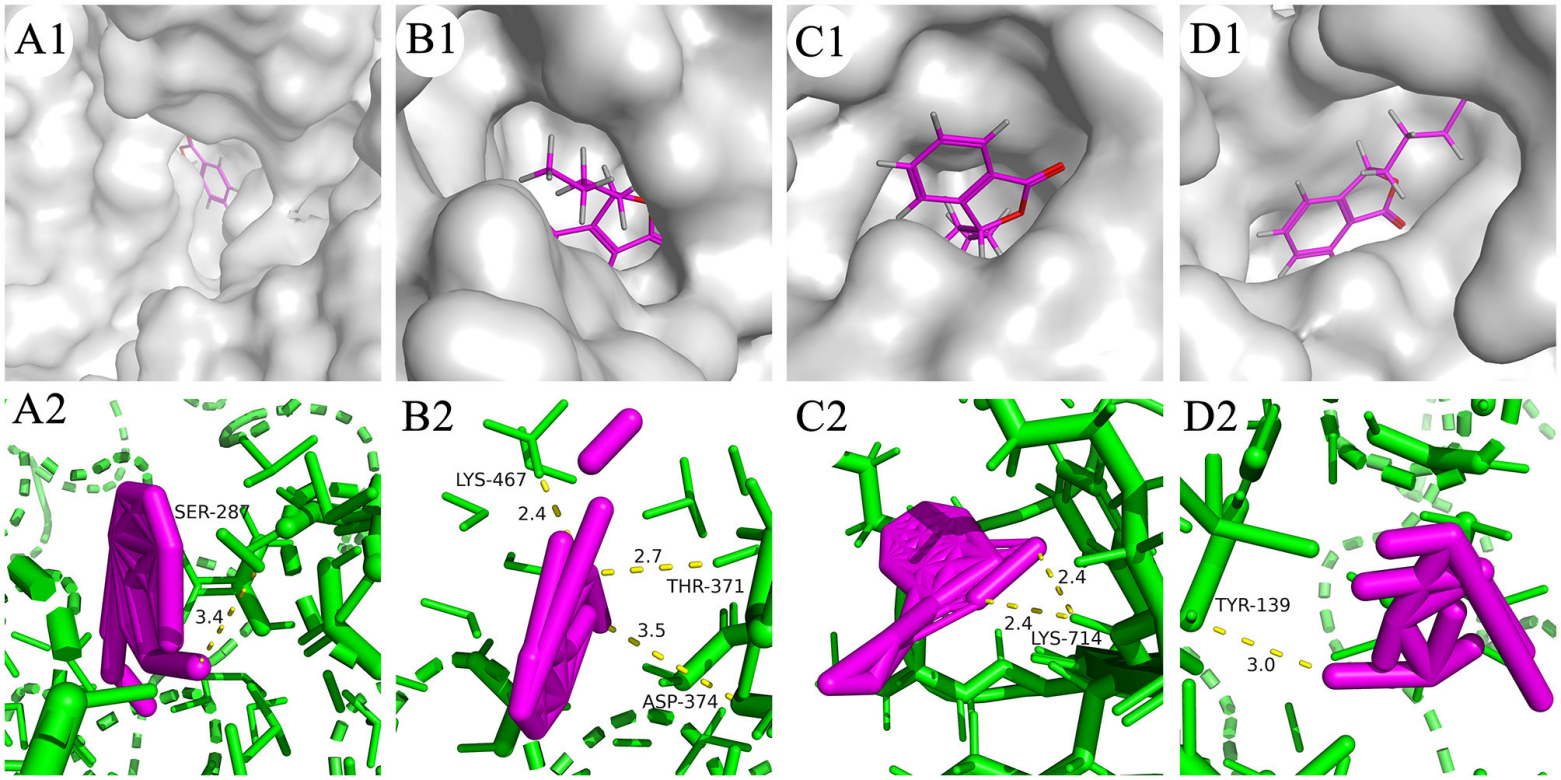
Docking model diagram. **(A)** *ALB*-NBP. **(B)** *ESR1*-NBP. **(C)** *EGFR*-NBP. **(D)** *HSP90AA1*-NBP. NBP, N-butylphthalide; ALB, albumin; ESR1, estrogen receptor 1; EGFR, epidermal growth factor receptor; HSP90AA1, Heat shock protein 90α.

Molecular docking was conducted between NBP and the key targets (*ALB, ESR1, EGFR, HSP90AA1*), the values of the docking energy represented the affinity of the components with the targets and the stability of the conformation. The binding energy which <0 indicated that the two molecules were able to bind spontaneously, the lower the binding energy the more stable the structure. The docking results showed that NBP could be bound into the docking pocket, with good docking activity between the target proteins (Figure 11). Table 3 shows the binding energy, all of them are <-5 kcal/mol, suggesting good docking activity. The docking results could provide data support for further relevant experimental designs in the future.

## 4. Discussion

### 4.1. Network meta-analysis

In the NMA, a total of 17 clinical RCTs were included and 1293 patients were enrolled, involving 16 interventions: acupuncture, CB-MNCs, nalmefene, MSCs + NBP, MSCs, kinnado + citicoline, citicoline, kinnado, XZYN, DXM, oxiracetam, hydrocortisone, tDCS, NBP + DXM, NBP, conventional therapy. This study not only included the latest relevant research so far, but also made up for the deficiency of a single comparison of traditional meta-analysis, and supplied more reference basis for the selection of clinical treatment regimens of DEACMP, it had certain clinical guiding significance. The results of this study showed that MSCs + NBP was the most effective in improving MMSE and BI scores; NBP + DXM was the most effective in improving ADL scores; NBP was the most effective in improving NIHSS scores; XZYN had more advantages in improving MoCA scores, tDCS had a significant effect in improving P300 latency and P300 amplitude, and kinnado + citicoline had the most obvious effect in improving MDA. DEACMP patients show different degrees of consciousness impairment, motor impairment, language dysfunction and memory impairment, while the above scales (MMSE, NIHSS, BI, ADL, MoCA) include exactly the evaluation of cognition, consciousness, language, motor, sensory, ataxic-motor and visual field, thus these scales were used to assess the change of patients' condition. Different patients exhibited different dysfunctions, and this study screened for regimens with better efficacy for each outcome indicator in order to provide a reference for clinical treatment. Limited by the number and quality of included studies, further validation with high-quality, multi-center, large-sample RCTs is recommended in the future.

NBP is widely used in the treatment of ischemic cerebrovascular diseases, and NBP can significantly improve the symptoms of neurological deficits (34). Previous studies (35–37) by our research team have demonstrated that NBP exhibits significant neuroprotective effects and is helpful in the treatment of acute carbon monoxide poisoning. These studies also revealed that NBP plays a multi-targeted role in protecting mitochondrial function, reducing neuronal apoptosis, resisting oxidative stress, maintaining the structural and functional integrity of the blood-brain barrier, and improving cognition. The NMA found that based on conventional therapy, NBP was the most effective in improving NIHSS scores, MSCs + NBP was the most effective in improving MMSE and BI scores, NBP + DXM was the most effective in improving ADL scores, it reveals that NBP may play a role in neuroprotection for patients with DEACMP based on conventional therapy.

### 4.2. Key targets

*ALB* can prevent platelet activation and aggregation, and also reduce apoptosis of vascular endothelial cells. In addition, *ALB* may be an indicator of atherosclerosis (38). Estrogen has numerous beneficial actions in the central nervous system, such as enhancing learning, memory, and stabilizing mood, as well as protection against a wide variety of noxious stimuli which may lead to neurodegenerative processes (39). Gene polymorphisms in the *ESR* can influence the expression of estrogen, thereby regulating different pathological processes. *EGFR* is distributed in all vascular epithelial cells, research has shown that *EGFR* could be transactivated during the phase of brain ischemia/reperfusion, which exerts neuroprotective effects (40). In eukaryotes, HSP90 is a well-characterized, conserved, and crucial chaperone protein. Not just the heat shock response, but several physiological processes are associated with the expression of *HSP90AA1*. It is related to the regulation of cell death, apoptosis, viral processing, and drug responses (41).

### 4.3. Main signaling pathway

The IL-17 family proteins are prominent and pleiotropic to regulate both pro-inflammatory and anti-inflammatory responses. In the aftermath of a stroke, IL-17 is critical in boosting the inflammatory response and causing secondary brain damage (42). Studies have demonstrated that the MAPK/ERK signaling pathway could enhance microcirculation, encourage angiogenesis, prevent cell apoptosis, and shield the brain tissue from toxicity effect of carbon monoxide (43). The FoxO signaling pathway plays an important role in cell proliferation, apoptosis, differentiation, and resistance to oxidative stress. Previous research works have demonstrated that it exerts a protective effect against neurodegenerative diseases not only by promoting apoptotic cell death in the nervous system but also by inducing autophagy (44). The PI3K/AKT signaling pathway is involved in the development and progression of various neurodegenerative diseases and plays an important role in cell proliferation, apoptosis, and oxidative stress (45). Estrogen activates endothelial nitric-oxide synthase (eNOS) *via* the PI3K/AKT pathway by the mediation of *ESR1* (46). *EGFR* can participate in the MAPK and FoxO signaling pathways, and *HSP90* is a target protein in the *IL-17* and *PI3K/AKT* signaling pathways. All the evidences stated above confirm indirectly that there is a close relationship between key targets and main pathways. Several of these signaling pathways have crosstalk effects, which are closely related to the pathological mechanism of DEACMP and the pharmacological mechanism of the drug.

## 5. Conclusion

The NMA screened for regimens with better efficacy for each outcome indicator in order to provide a reference for clinical treatment, but limited by the number and quality of included studies, further validation with high-quality, multi-center, large-sample RCTs is recommended in the future. NBP can stably bind *ALB, ESR1, EGFR, HSP90AA1*, and other targets, and it may play a role in neuroprotection for patients with DEACMP by modulating Lipid and atherosclerosis, *IL-17* signaling pathway, *MAPK* signaling pathway, *FoxO* signaling pathway, *PI3K/AKT* signaling pathway.

In summary, this study screened for regimens with better efficacy for each outcome indicator and provided a reference for clinical treatment, and then predicted the pharmacological mechanism of NBP from a systematic perspective based on network pharmacology and molecular docking techniques. We found that NBP can stably bind *ALB, ESR1, EGFR, HSP90AA1*, and other targets, and it is involved in the cellular response to lipids, response to hormone, enzyme-linked receptor protein signaling pathway, protein phosphorylation, transmembrane receptor protein tyrosine kinase signaling pathway, and other important biological processes through the regulation of Lipid and atherosclerosis, *IL-17* signaling pathway, *MAPK* signaling pathway, *FoxO* signaling pathway, *PI3K/AKT* signaling pathway, which plays a significant role in the treatment of DEACMP.

## Data availability statement

The original contributions presented in the study are included in the article/Supplementary material, further inquiries can be directed to the corresponding author/s.

## Author contributions

Conception of the study: HS and QL. Entering data and analyzing and interpreting data: HS, AY, and XZ. Manuscript preparation: HS and AY. Critically revising the article: HS, QL, and WH. Administrative, technical, and material support: WH. All authors contributed to the article and approved the submitted version.
