# Supplementary material for: Evidence of clinical efficacy and pharmacological mechanism of N-butylphthalide in the treatment of delayed encephalopathy after acute carbon monoxide poisoning
Source: Front Neurol. 2023 Mar 16;14:1119871. doi: 10.3389/fneur.2023.1119871 (PMC10060646; doi:10.3389/fneur.2023.1119871)
Supplement: Supplementary file 1 [file Data_Sheet_1.ZIP › Network meta-analysis/search records.doc]

Pubmed检索式

- (((((((((((((delayed encephalopathy after acute carbon monoxide poisoning[Title/Abstract]) OR (delayed encephalopathy[Title/Abstract])) OR (carbon monoxide intoxication[Title/Abstract])) OR (Poisoning, Carbon Monoxide[Title/Abstract])) OR (Carbon Monoxide Poisonings[Title/Abstract])) OR (Monoxide Poisoning, Carbon[Title/Abstract])) OR (Monoxide Poisonings, Carbon[Title/Abstract])) OR (Poisonings, Carbon Monoxide[Title/Abstract])) OR (Poisoning, Illuminating Gas[Title/Abstract])) OR (Gas Poisoning, Illuminating[Title/Abstract])) OR (Gas Poisonings, Illuminating[Title/Abstract])) OR (Illuminating Gas Poisonings[Title/Abstract])) OR (Poisonings, Illuminating Gas[Title/Abstract])) OR (Illuminating Gas Poisoning[Title/Abstract])OR (ACOP[Title/Abstract])) OR (DEACMP[Title/Abstract])AND((((((((((((((((((((((((((((((((((((((((((((((((((((((((((((((((((((((((((((((((((((((((((((((((((((((((((((((((hyperbaric oxygen[Title/Abstract]) OR (Hyperbaric Oxygenations[Title/Abstract])) OR (Oxygenations, Hyperbaric[Title/Abstract])) OR (Hyperbaric Oxygen Therapy[Title/Abstract])) OR (Hyperbaric Oxygen Therapies[Title/Abstract])) OR (Oxygen Therapies, Hyperbaric[Title/Abstract])) OR (Oxygen Therapy, Hyperbaric[Title/Abstract])) OR (Therapies, Hyperbaric Oxygen[Title/Abstract])) OR (Therapy, Hyperbaric Oxygen[Title/Abstract])) OR (Oxygenation, Hyperbaric[Title/Abstract])) OR (3-n-butylphthalide[Title/Abstract])) OR (l-NBP cpd[Title/Abstract])) OR (N-butylphthalide[Title/Abstract])) OR (butylphthalide[Title/Abstract])) OR ((S)-(-)-3-butylphthalide[Title/Abstract])) OR (Glucocorticoids[Title/Abstract])) OR (Glucocorticoids[Title/Abstract])) OR (Glucocorticoid Effect[Title/Abstract])) OR (Effect, Glucocorticoid[Title/Abstract])) OR (Glucorticoid Effects[Title/Abstract])) OR (Effects, Glucorticoid[Title/Abstract])) OR (Dexamethasone[Title/Abstract])) OR (Methylfluorprednisolone[Title/Abstract])) OR (Hexadecadrol[Title/Abstract])) OR (Decameth[Title/Abstract])) OR (Decaspray[Title/Abstract])) OR (Dexasone[Title/Abstract])) OR (Dexpak[Title/Abstract])) OR (Maxidex[Title/Abstract])) OR (Millicorten[Title/Abstract])) OR (Oradexon[Title/Abstract])) OR (Decaject[Title/Abstract])) OR (Decaject-L.A.[Title/Abstract])) OR (Decaject L.A.[Title/Abstract])) OR (Hexadrol[Title/Abstract])) OR (oxiracetam[Title/Abstract])) OR (ISF 2522[Title/Abstract])) OR (ISF-2522[Title/Abstract])) OR (Kinnado[Title/Abstract])) OR (Ginaton[Title/Abstract])) OR (Ginkgo biloba[Title/Abstract])) OR (Ginkgo bilobas[Title/Abstract])) OR (bilobas, Ginkgo[Title/Abstract])) OR (Gingko[Title/Abstract])) OR (Gingkos[Title/Abstract])) OR (Gingko biloba[Title/Abstract])) OR (Gingko bilobas[Title/Abstract])) OR (bilobas, Gingko[Title/Abstract])) OR (Ginkgo[Title/Abstract])) OR (Ginkgos[Title/Abstract])) OR (Ginko[Title/Abstract])) OR (Ginkos[Title/Abstract])) OR (Maidenhair Tree[Title/Abstract])) OR (Maidenhair Trees[Title/Abstract])) OR (Tree, Maidenhair[Title/Abstract])) OR (Trees, Maidenhair[Title/Abstract])) OR (Ginkgophyta[Title/Abstract])) OR (Ginkgophytas[Title/Abstract])) OR (ganglioside[Title/Abstract])) OR (gangliosides[Title/Abstract])) OR (Sialoglycosphingolipids[Title/Abstract])) OR (nalmefene[Title/Abstract])) OR (Revex[Title/Abstract])) OR (Selincro[Title/Abstract])) OR (6-desoxy-6-methylenenaltrexone[Title/Abstract])) OR (nalmefene hydrochloride[Title/Abstract])) OR (XingZhi-YiNao granules[Title/Abstract])) OR (umbilical cord blood mononuclear cells[Title/Abstract])) OR (umbilical cord blood mononuclear cell[Title/Abstract])) OR (umbilical mesenchymal stem cells[Title/Abstract])) OR (umbilical mesenchymal stem cell[Title/Abstract])) OR (Acupuncture[Title/Abstract])) OR (Pharmacopuncture[Title/Abstract])) OR (transcranial direct current stimulation[Title/Abstract])) OR (tDCS[Title/Abstract])) OR (Cathodal Stimulation Transcranial Direct Current Stimulation[Title/Abstract])) OR (Cathodal Stimulation tDCS[Title/Abstract])) OR (Cathodal Stimulation tDCSs[Title/Abstract])) OR (Stimulation tDCS, Cathodal[Title/Abstract])) OR (Stimulation tDCSs, Cathoda[Title/Abstract])) OR (tDCS, Cathodal Stimulation[Title/Abstract])) OR (tDCSs, Cathodal Stimulation[Title/Abstract])) OR (Transcranial Random Noise Stimulation[Title/Abstract])) OR (Transcranial Alternating Current Stimulation[Title/Abstract])) OR (Transcranial Electrical Stimulation[Title/Abstract])) OR (Electrical Stimulation, Transcranial[Title/Abstract])) OR (Electrical Stimulations, Transcranial[Title/Abstract])) OR (Stimulation, Transcranial Electrical[Title/Abstract])) OR (Stimulations, Transcranial Electrical[Title/Abstract])) OR (Transcranial Electrical Stimulations[Title/Abstract])) OR (Anodal Stimulation Transcranial Direct Current Stimulation[Title/Abstract])) OR (Anodal Stimulation tDCS[Title/Abstract])) OR (Anodal Stimulation tDCSs[Title/Abstract])) OR (Stimulation tDCS, Anodal[Title/Abstract])) OR (Stimulation tDCSs, Anodal[Title/Abstract])) OR (tDCS, Anodal Stimulation[Title/Abstract])) OR (tDCSs, Anodal Stimulation[Title/Abstract])) OR (Repetitive Transcranial Electrical Stimulation[Title/Abstract])) OR (Edaravone[Title/Abstract])) OR (Norantipyrine[Title/Abstract])) OR (Norphenazone[Title/Abstract])) OR (Edarabone[Title/Abstract])) OR (Radicava[Title/Abstract])) OR (Phenylmethylpyrazolone[Title/Abstract])) OR (citicoline[Title/Abstract])) OR (Cytidine Diphosphate[Title/Abstract])) OR (Citicoline[Title/Abstract])) OR (Citicholine[Title/Abstract])) OR (CDP Choline[Title/Abstract])) OR (nerve growth factor[Title/Abstract])) OR (NGF[Title/Abstract])) OR (Erythropoietin[Title/Abstract])) OR (EPO[Title/Abstract])

EMBSAE

‘delayed encephalopathy after acute carbon monoxide poisoning’:ab,ti OR ‘delayed encephalopathy’:ab,ti OR ‘carbon monoxide intoxication’:ab,ti OR ‘Poisoning,Carbon Monoxide’:ab,ti OR ‘Carbon Monoxide Poisonings’:ab,ti OR ‘Monoxide Poisoning, Carbon’:ab,ti OR ‘Monoxide Poisonings,Carbon’:ab,ti OR ‘Poisonings,Carbon Monoxide’:ab,ti OR ‘Poisoning,Illuminating Gas’:ab,ti OR ‘Gas Poisoning, Illuminating’:ab,ti OR ‘Gas Poisonings, Illuminating’:ab,ti OR ‘Illuminating Gas Poisonings’:ab,ti OR ‘Poisonings,Illuminating Gas’:ab,ti OR ‘Illuminating Gas Poisoning’:ab,ti OR ‘ACOP’:ab,ti OR ‘DEACMP’:ab,ti

(delayed encephalopathy after acute carbon monoxide poisoning or delayed encephalopathy or carbon monoxide intoxication or Poisoning, Carbon Monoxide or Monoxide Poisoning, Carbon or Monoxide Poisonings, Carbon or Gas Poisoning, Illuminating or Gas Poisonings, Illuminating or Illuminating Gas Poisonings or Poisonings, Illuminating Gas or Illuminating Gas Poisoning or ACOP or DEACMP or Carbon Monoxide Poisonings or Poisonings, Carbon Monoxide).ab.

'delayed encephalopathy after acute carbon monoxide poisoning':ab,ti OR 'delayed encephalopathy':ab,ti OR 'carbon monoxide intoxication':ab,ti OR 'Poisoning, Carbon Monoxide':ab,ti OR 'Carbon Monoxide Poisonings':ab,ti OR 'Monoxide Poisoning, Carbon':ab,ti OR 'Monoxide Poisonings, Carbon':ab,ti OR 'Poisonings, Carbon Monoxide':ab,ti OR 'Poisoning, Illuminating Gas':ab,ti OR 'Gas Poisoning, Illuminating':ab,ti OR 'Gas Poisonings, Illuminating':ab,ti OR 'Illuminating Gas Poisonings':ab,ti OR 'Poisonings, Illuminating Gas':ab,ti OR 'Illuminating Gas Poisoning':ab,ti OR 'ACOP':ab,ti OR 'DEACMP':ab,ti

‘hyperbaric oxygen’:ab,ti OR ‘Hyperbaric Oxygenations’:ab,ti OR ‘Oxygenations, Hyperbaric’:ab,ti OR ‘Hyperbaric Oxygen Therapy’:ab,ti OR ‘Hyperbaric Oxygen Therapies’:ab,ti OR ‘Oxygen Therapies, Hyperbaric’:ab,ti OR ‘Oxygen Therapy, Hyperbaric’:ab,ti OR ‘Therapies, Hyperbaric Oxygen’:ab,ti OR ‘Therapy, Hyperbaric Oxygen’:ab,ti OR ‘Oxygenation, Hyperbaric’:ab,ti OR ‘3-n-butylphthalide’:ab,ti OR ‘3-n-butylphthalide’:ab,ti OR ‘N-butylphthalide’:ab,ti OR ‘butylphthalide’:ab,ti OR ‘(S)-(-)-3-butylphthalide’:ab,ti OR ‘Glucocorticoids’:ab,ti OR ‘Glucocorticoids’:ab,ti OR ‘Glucocorticoid Effect’:ab,ti OR ‘Effect, Glucocorticoid’:ab,ti OR ‘Glucorticoid Effects’:ab,ti OR ‘Effects, Glucorticoid’:ab,ti OR ‘Dexamethasone’:ab,ti OR ‘Methylfluorprednisolone’:ab,ti OR ‘Hexadecadrol’:ab,ti OR ‘Decameth’:ab,ti OR ‘Decaspray’:ab,ti OR ‘Dexasone’:ab,ti OR ‘Dexpak’:ab,ti OR ‘Maxidex’:ab,ti OR ‘Millicorten’:ab,ti OR ‘Oradexon’:ab,ti OR ‘Decaject’:ab,ti OR ‘Decaject-L.A.’:ab,ti OR ‘Decaject L.A.’:ab,ti OR ‘Hexadrol’:ab,ti OR ‘oxiracetam’:ab,ti OR ‘ISF 2522’:ab,ti OR ‘ISF-2522’:ab,ti OR ‘Kinnado’:ab,ti OR ‘Ginaton’:ab,ti OR ‘Ginkgo biloba’:ab,ti OR ‘Ginkgo bilobas’:ab,ti OR ‘bilobas, Ginkgo’:ab,ti OR ‘Gingko’:ab,ti OR ‘Gingkos’:ab,ti OR ‘Gingko biloba’:ab,ti OR ‘Gingko bilobas’:ab,ti OR ‘bilobas, Gingko’:ab,ti OR ‘Ginkgo’:ab,ti OR ‘Ginkgos’:ab,ti OR ‘Ginko’:ab,ti OR ‘Ginkos’:ab,ti OR ‘Maidenhair Tree’:ab,ti OR ‘Maidenhair Trees’:ab,ti OR ‘Tree, Maidenhair’:ab,ti OR ‘Trees, Maidenhair’:ab,ti OR ‘Ginkgophyta’:ab,ti OR ‘Ginkgophytas’:ab,ti OR ‘ganglioside’:ab,ti OR ‘gangliosides’:ab,ti OR ‘Sialoglycosphingolipids’:ab,ti OR ‘nalmefene’:ab,ti OR ‘Revex’:ab,ti OR ‘Selincro’:ab,ti OR ‘6-desoxy-6-methylenenaltrexone’:ab,ti OR ‘nalmefene hydrochloride’:ab,ti OR ‘XingZhi-YiNao granules’:ab,ti OR ‘umbilical cord blood mononuclear cells’:ab,ti OR ‘umbilical cord blood mononuclear cell’:ab,ti OR ‘umbilical mesenchymal stem cells’:ab,ti OR ‘umbilical mesenchymal stem cell’:ab,ti OR ‘Acupuncture’:ab,ti OR ‘Pharmacopuncture’:ab,ti OR ‘transcranial direct current stimulation’:ab,ti OR ‘tDCS’:ab,ti OR ‘Cathodal Stimulation Transcranial Direct Current Stimulation’:ab,ti OR ‘Cathodal Stimulation tDCS’:ab,ti OR ‘Cathodal Stimulation tDCSs’:ab,ti OR ‘Stimulation tDCS, Cathodal’:ab,ti OR ‘Stimulation tDCSs, Cathoda’:ab,ti OR ‘Stimulation tDCSs, Cathoda’:ab,ti OR ‘tDCSs, Cathodal Stimulation’:ab,ti OR ‘Transcranial Random Noise Stimulation’:ab,ti OR ‘Transcranial Alternating Current Stimulation’:ab,ti OR ‘Transcranial Electrical Stimulation’:ab,ti OR ‘Electrical Stimulation, Transcranial’:ab,ti OR ‘Electrical Stimulations, Transcranial’:ab,ti OR ‘Stimulation, Transcranial Electrical’:ab,ti OR ‘Stimulation, Transcranial Electrical’:ab,ti OR ‘Transcranial Electrical Stimulations’:ab,ti OR ‘Anodal Stimulation Transcranial Direct Current Stimulation’:ab,ti OR ‘Anodal Stimulation tDCS’:ab,ti OR ‘Anodal Stimulation tDCSs’:ab,ti OR ‘Stimulation tDCS, Anodal’:ab,ti OR ‘Stimulation tDCSs, Anodal’:ab,ti OR ‘tDCS, Anodal Stimulation’:ab,ti OR ‘tDCSs, Anodal Stimulation’:ab,ti OR ‘Repetitive Transcranial Electrical Stimulation’:ab,ti

Cochrane

delayed encephalopathy after acute carbon monoxide poisoning OR delayed encephalopathy OR carbon monoxide intoxication OR Poisoning, Carbon Monoxide OR Carbon Monoxide Poisonings OR Monoxide Poisoning, Carbon OR Monoxide Poisonings, Carbon OR Poisonings, Carbon Monoxide OR Poisoning, Illuminating Gas OR Gas Poisoning, Illuminating OR Gas Poisonings, Illuminating OR Illuminating Gas Poisonings OR Poisonings, Illuminating Gas OR Illuminating Gas Poisoning OR ACOP OR DEACMP

(S)-(-)-3-butylphthalide	OR 3-n-butylphthalide OR 3-n-butylphthalide

hyperbaric oxygen OR Hyperbaric Oxygenations OR Oxygenations, Hyperbaric OR Hyperbaric Oxygen Therapy OR Hyperbaric Oxygen Therapies OR Oxygen Therapies, Hyperbaric OR Oxygen Therapy, Hyperbaric OR Therapies, Hyperbaric Oxygen OR Therapy, Hyperbaric Oxygen OR Oxygenation, Hyperbaric OR N-butylphthalide OR butylphthalide OR Glucocorticoids OR Glucocorticoids OR Glucocorticoid Effect OR Effect, Glucocorticoid OR Glucorticoid Effects OR Effects, Glucorticoid OR Dexamethasone OR Methylfluorprednisolone OR Hexadecadrol OR Decameth OR Decaspray OR Dexasone OR Dexpak OR Maxidex OR Millicorten OR Oradexon OR Decaject OR Decaject-L.A. OR Decaject L.A. OR Hexadrol OR oxiracetam OR ISF 2522 OR ISF-2522 OR Kinnado OR Ginaton OR Ginkgo biloba OR Ginkgo bilobas OR bilobas, Ginkg OR Gingko OR Gingkos OR Gingko biloba OR Gingko bilobas OR bilobas, Gingko OR Ginkgo OR Ginkgos OR Ginko OR Ginkos OR Ginkgophyta OR Ginkgophytas OR ganglioside OR gangliosides OR Sialoglycosphingolipids OR nalmefene OR Revex OR Selincro OR nalmefene hydrochloride OR XingZhi-YiNao granules OR umbilical cord blood mononuclear cells OR umbilical cord blood mononuclear cell OR umbilical mesenchymal stem cells OR umbilical mesenchymal stem cell OR Acupuncture OR Pharmacopuncture OR transcranial direct current stimulation OR tDCS OR Cathodal Stimulation Transcranial Direct Current Stimulation OR Cathodal Stimulation tDCS OR Cathodal Stimulation tDCSs OR Stimulation tDCS, Cathodal OR Stimulation tDCSs, Cathoda OR Stimulation tDCSs, Cathoda OR tDCSs, Cathodal Stimulation OR Transcranial Random Noise Stimulation OR Transcranial Alternating Current Stimulation OR Transcranial Electrical Stimulation OR Electrical Stimulation, Transcranial OR Electrical Stimulations, Transcranial OR Stimulation, Transcranial Electrical OR Stimulation, Transcranial Electrical OR Transcranial Electrical Stimulations OR Anodal Stimulation Transcranial Direct Current Stimulation OR Anodal Stimulation tDCS OR Anodal Stimulation tDCSs OR Stimulation tDCS, Anodal OR Stimulation tDCSs, Anodal OR tDCS, Anodal Stimulation OR tDCSs, Anodal Stimulation OR Repetitive Transcranial Electrical Stimulation OR Edaravone OR Norantipyrine OR Norphenazone OR Edarabone OR Radicava OR Phenylmethylpyrazolone OR citicoline OR Cytidine Diphosphate OR Citicoline OR Citicholine OR CDP Choline OR nerve growth factor OR NGF OR Erythropoietin OR EPO

Web of science

TS=(delayed encephalopathy after acute carbon monoxide poisoning OR delayed encephalopathy OR carbon monoxide intoxication OR Poisoning, Carbon Monoxide OR Carbon Monoxide Poisonings OR Monoxide Poisoning, Carbon OR Monoxide Poisonings, Carbon OR Poisonings, Carbon Monoxide OR Poisoning, Illuminating Gas OR Gas Poisoning, Illuminating OR Gas Poisonings, Illuminating OR Illuminating Gas Poisonings OR Poisonings, Illuminating Gas OR Illuminating Gas Poisoning OR ACOP OR DEACMP)

AB=(hyperbaric oxygen OR Hyperbaric Oxygenations OR Oxygenations, Hyperbaric OR Hyperbaric Oxygen Therapy OR Hyperbaric Oxygen Therapies OR Oxygen Therapies, Hyperbaric OR Oxygen Therapy, Hyperbaric OR Therapies, Hyperbaric Oxygen OR Therapy, Hyperbaric Oxygen OR Oxygenation, Hyperbaric OR N-butylphthalide OR butylphthalide OR Glucocorticoids OR Glucocorticoids OR Glucocorticoid Effect OR Effect, Glucocorticoid OR Glucorticoid Effects OR Effects, Glucorticoid OR Dexamethasone OR Methylfluorprednisolone OR Hexadecadrol OR Decameth OR Decaspray OR Dexasone OR Dexpak OR Maxidex OR Millicorten OR Oradexon OR Decaject OR Decaject-L.A. OR Decaject L.A. OR Hexadrol OR oxiracetam OR ISF 2522 OR ISF-2522 OR Kinnado OR Ginaton OR Ginkgo biloba OR Ginkgo bilobas OR bilobas, Ginkg OR Gingko OR Gingkos OR Gingko biloba OR Gingko bilobas OR bilobas, Gingko OR Ginkgo OR Ginkgos OR Ginko OR Ginkos OR Ginkgophyta OR Ginkgophytas OR ganglioside OR gangliosides OR Sialoglycosphingolipids OR nalmefene OR Revex OR Selincro OR nalmefene hydrochloride OR XingZhi-YiNao granules OR umbilical cord blood mononuclear cells OR umbilical cord blood mononuclear cell OR umbilical mesenchymal stem cells OR umbilical mesenchymal stem cell OR Acupuncture OR Pharmacopuncture OR transcranial direct current stimulation OR tDCS OR Cathodal Stimulation Transcranial Direct Current Stimulation OR Cathodal Stimulation tDCS OR Cathodal Stimulation tDCSs OR Stimulation tDCS, Cathodal OR Stimulation tDCSs, Cathoda OR Stimulation tDCSs, Cathoda OR tDCSs, Cathodal Stimulation OR Transcranial Random Noise Stimulation OR Transcranial Alternating Current Stimulation OR Transcranial Electrical Stimulation OR Electrical Stimulation, Transcranial OR Electrical Stimulations, Transcranial OR Stimulation, Transcranial Electrical OR Stimulation, Transcranial Electrical OR Transcranial Electrical Stimulations OR Anodal Stimulation Transcranial Direct Current Stimulation OR Anodal Stimulation tDCS OR Anodal Stimulation tDCSs OR Stimulation tDCS, Anodal OR Stimulation tDCSs, Anodal OR tDCS, Anodal Stimulation OR tDCSs, Anodal Stimulation OR Repetitive Transcranial Electrical Stimulation OR Edaravone OR Norantipyrine OR Norphenazone OR Edarabone OR Radicava OR Phenylmethylpyrazolone OR citicoline OR Cytidine Diphosphate OR Citicoline OR Citicholine OR CDP Choline OR nerve growth factor OR NGF OR Erythropoietin OR EPO)

Scopus

Effects of butyphthalide and edaravone on diffusion tensor imaging and electroencephalogram on acute carbon monoxide poisoning delayed encephalopathy.

EBM Reviews - ACP Journal Club <1991 to August 2021>

EBM Reviews - Cochrane Central Register of Controlled Trials <August 2021>

EBM Reviews - Cochrane Database of Systematic Reviews <2005 to September 9, 2021>

EBM Reviews - Cochrane Clinical Answers <August 2021>

EBM Reviews - Cochrane Methodology Register <3rd Quarter 2012>

EBM Reviews - Database of Abstracts of Reviews of Effects <1st Quarter 2016>

EBM Reviews - Health Technology Assessment <4th Quarter 2016>

EBM Reviews - NHS Economic Evaluation Database <1st Quarter 2016>

Embase <1974 to 2021 September 14>

1	delayed encephalopathy after acute carbon monoxide poisoning.mp. [mp=ti, ot, ab, tx, kw, ct, sh, hw, tn, dm, mf, dv, kf, fx, dq]	72

2	delayed encephalopathy.mp. [mp=ti, ot, ab, tx, kw, ct, sh, hw, tn, dm, mf, dv, kf, fx, dq]	239

3	carbon monoxide intoxication.mp. [mp=ti, ot, ab, tx, kw, ct, sh, hw, tn, dm, mf, dv, kf, fx, dq]	5541

4	Poisoning, Carbon Monoxide.mp. [mp=ti, ot, ab, tx, kw, ct, sh, hw, tn, dm, mf, dv, kf, fx, dq]	29

5	Carbon Monoxide Poisonings.mp. [mp=ti, ot, ab, tx, kw, ct, sh, hw, tn, dm, mf, dv, kf, fx, dq]	74

6	Monoxide Poisoning, Carbon.mp. [mp=ti, ot, ab, tx, kw, ct, sh, hw, tn, dm, mf, dv, kf, fx, dq]	3

7	Monoxide Poisonings, Carbon.mp. [mp=ti, ot, ab, tx, kw, ct, sh, hw, tn, dm, mf, dv, kf, fx, dq]	0

8	Poisonings, Carbon Monoxide.mp. [mp=ti, ot, ab, tx, kw, ct, sh, hw, tn, dm, mf, dv, kf, fx, dq]	1

9	Poisoning, Illuminating Gas.mp. [mp=ti, ot, ab, tx, kw, ct, sh, hw, tn, dm, mf, dv, kf, fx, dq]	0

10	Gas Poisoning, Illuminating.mp. [mp=ti, ot, ab, tx, kw, ct, sh, hw, tn, dm, mf, dv, kf, fx, dq]	0

11	Gas Poisonings, Illuminating.mp. [mp=ti, ot, ab, tx, kw, ct, sh, hw, tn, dm, mf, dv, kf, fx, dq]	0

12	Illuminating Gas Poisonings.mp. [mp=ti, ot, ab, tx, kw, ct, sh, hw, tn, dm, mf, dv, kf, fx, dq]	0

13	Poisonings, Illuminating Gas.mp. [mp=ti, ot, ab, tx, kw, ct, sh, hw, tn, dm, mf, dv, kf, fx, dq]	0

14	Illuminating Gas Poisoning.mp. [mp=ti, ot, ab, tx, kw, ct, sh, hw, tn, dm, mf, dv, kf, fx, dq]	0

15	ACOP.mp. [mp=ti, ot, ab, tx, kw, ct, sh, hw, tn, dm, mf, dv, kf, fx, dq]	120

16	DEACMP.mp. [mp=ti, ot, ab, tx, kw, ct, sh, hw, tn, dm, mf, dv, kf, fx, dq]	67

17	1 or 2 or 3 or 4 or 5 or 6 or 7 or 8 or 9 or 10 or 11 or 12 or 13 or 14 or 15 or 16	5729

18	treatment.mp. [mp=ti, ot, ab, tx, kw, ct, sh, hw, tn, dm, mf, dv, kf, fx, dq]	8349288

19	17 and 18	1445
